# 1150. Invasive meningococcal disease vaccination – a targeted literature review of adolescents and parents/caregivers’ preferences

**DOI:** 10.1093/ofid/ofad500.991

**Published:** 2023-11-27

**Authors:** Shahina Begum, Eliazar Sabater Cabrera, Oscar Herrera-Restrepo, Twinkle Khera, Willings Botha, Laurie Batchelder, Zeki Kocaata

**Affiliations:** GSK, London, England, United Kingdom; GSK, London, England, United Kingdom; GSK, London, England, United Kingdom; IQVIA, Bangalore, Karnataka, India; IQVIA, Bangalore, Karnataka, India; IQVIA, Bangalore, Karnataka, India; GSK, London, England, United Kingdom

## Abstract

**Background:**

Invasive meningococcal disease (IMD) serogroups A, B, C, W, Y are commonly prevented by MenACWY and MenB vaccines. MenABCWY candidate vaccines could potentially provide benefits as less injections, simplified schedules, and increased uptake. However, there is limited insight on factors influencing preferences for IMD vaccines/vaccination (Vax). This targeted literature review synthesized evidence of factors influencing IMD Vax preferences in 16–23-year-old adolescents/young adults (Ado/YA) and parents/caregivers (P/CG) of 16-18 year-old adolescents.

**Methods:**

PubMed and Google Scholar were searched globally to identify publications on IMD Vax attitudes and preferences (Table 1). Studies were restricted to English and published between 2005-2022. Data were extracted and synthesized from full text reviews to list IMD Vax preference attributes.

**Results:**

From the 77 abstracts screened, 19 publications were extracted (Table 2) and 17 relevant for Ado/YA and P/CG. Knowledge of disease severity (20% of Ado/YA articles) and vaccine (29% of P/CG articles) were the most reported factors influencing Vax preference. Severity of disease increased Vax preference for both groups (14%), while low disease awareness limited P/CGs’ willingness to vaccinate children (14% of P/CG articles). Some Ado/YA preferred fewer injections in the immunization series due to reduced injection site discomforts (13%). P/CG preferred less injections due to less time and less physician visits, as it may reduce vaccine preparation/injection/administration and indirect costs associated with parental work loss (7%). However, their concerns over injection-related pain were a Vax barrier (14%). IMD vaccine effectiveness was recognized by Ado/YA (13%). Longer duration of protection was important for P/CG (14%), whilst herd immunity and direct protection was preferred in Ado/YA (13%).

**Figure:**
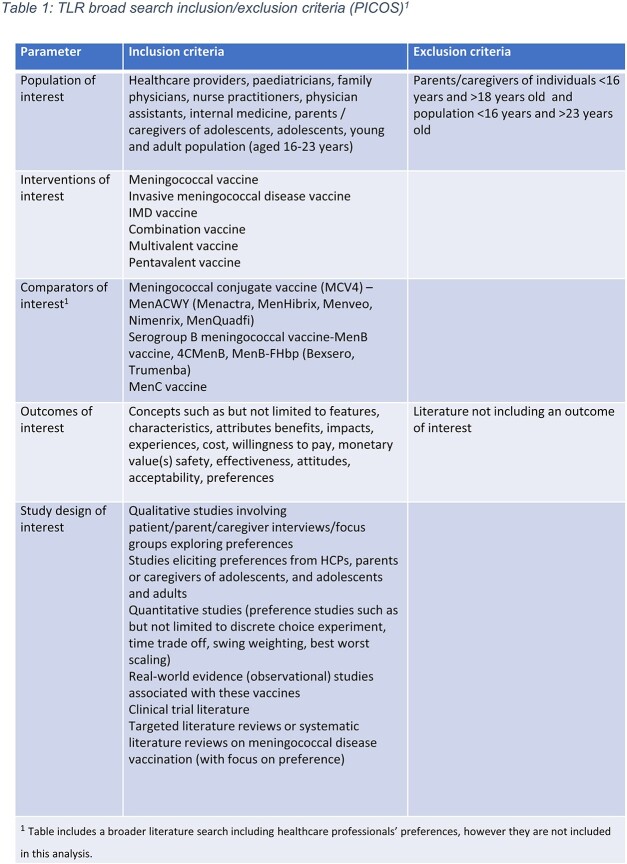

**Figure:**
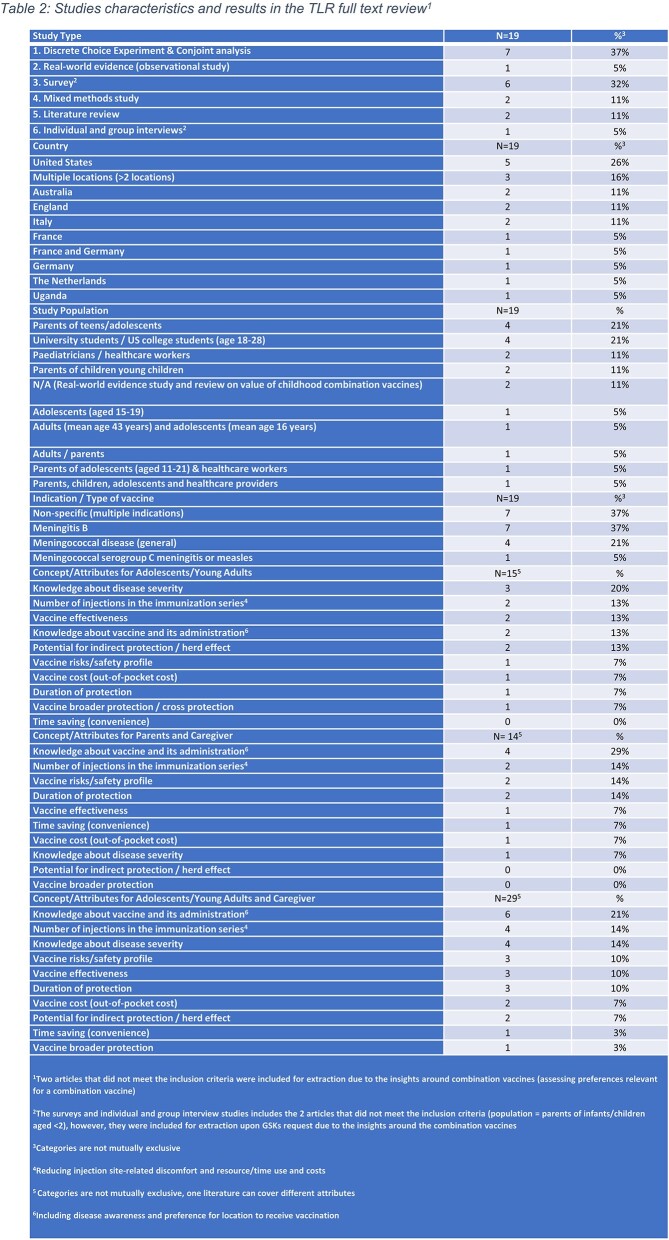

**Conclusion:**

Findings highlight IMD Vax characteristics as key considerations among Ado/YA and P/CG when making Vax decisions. To improve vaccination coverage and protection, the evidence supports preferences for vaccinations offering benefits such as fewer injections. Trade-offs between factors relevant for a IMD combination vaccine need further research.

**Disclosures:**

**Shahina Begum**, GSK: Employee **Eliazar Sabater Cabrera, PhD**, GSK: Employee|GSK: Stocks/Bonds **Oscar Herrera-Restrepo, PhD**, GSK: Stocks/Bonds **Twinkle Khera, Mtech**, IQVIA: Advisor/Consultant **Willings Botha, PhD**, IQVIA: Advisor/Consultant **Laurie Batchelder, PhD**, IQVIA: Advisor/Consultant **Zeki Kocaata, PhD**, GSK: Stocks/Bonds

